# Effects of cognitive behavioural therapy for depression in heart failure patients: a systematic review and meta-analysis

**DOI:** 10.1007/s10741-017-9640-5

**Published:** 2017-07-22

**Authors:** Kishaan Jeyanantham, Dipak Kotecha, Devsaagar Thanki, Rebecca Dekker, Deirdre A. Lane

**Affiliations:** 1 The Medical School, University of Birmingham, Edgbaston, Birmingham, B15 2TT UK; 2 University of Birmingham Institute of Cardiovascular Sciences, City Hospital, Sandwell and West Birmingham Hospitals NHS Trust, Dudley Road, Birmingham, B18 7QH UK; 3 Monash University Centre of Cardiovascular Research and Education in Therapeutics, Melbourne, Australia; 4 University of Kentucky, Lexington, KY USA; 5 Aalborg Thrombosis Research Unit, Department of Clinical Medicine, Aalborg University, Aalborg, Denmark

**Keywords:** Cognitive behavioural therapy, Depression, Heart failure, Meta-analysis, Quality of life, Systematic review

## Abstract

This systematic review and meta-analysis aimed to evaluate the effects of cognitive behavioural therapy (CBT) on depression, quality of life, hospitalisations and mortality in heart failure patients. The search strategy was developed for Ovid MEDLINE and modified accordingly to search the following bibliographic databases: PubMed, EMBASE, PsycINFO, CENTRAL and CINAHL. Databases were searched from inception to 6 March 2016 for randomised controlled trials (RCTs) or observational studies that used CBT in heart failure patients with depression or depressive symptoms. Six studies were identified: 5 RCTs and 1 observational study, comprising 320 participants with predominantly NYHA classes II-III, who were mostly male, with mean age ranging from 55 to 66 years. Compared to usual care, CBT was associated with a greater improvement in depression scores both initially after CBT sessions (standardised mean difference −0.34, 95% CI −0.60 to −0.08, *p* = 0.01) and at 3 months follow-up (standardised mean difference −0.32, 95% CI −0.59 to −0.04, *p* = 0.03). Greater improvement in quality of life scores was evident for the CBT group initially after CBT sessions, but with no difference at 3 months. Hospital admissions and mortality were similar, regardless of treatment group. CBT may be more effective than usual care at improving depression scores and quality of life for heart failure patients initially following CBT and for depression at 3 months. Larger and more robust RCTs are needed to evaluate the long-term clinical effects of CBT in heart failure patients.

**Electronic supplementary material:**

The online version of this article (doi:10.1007/s10741-017-9640-5) contains supplementary material, which is available to authorized users.

## Introduction

Heart failure continues to impose a tremendous burden on patients, carers and healthcare systems. In Europe, approximately 6.5 million people are currently living with the condition [1], with a prevalence of ≥10% among the 70 years and older population [2]. Heart failure is the endpoint of all cardiovascular diseases [3], and therefore, the improved survival rates for other cardiovascular diseases are expected to further increase the prevalence, in addition to the increase due to the ageing population [2]. It is a significant cause of mortality, with approximately 5% of all deaths attributable to heart failure in the UK [4], and only 25% of patients are expected to survive beyond 5 years after their first hospital admission [5]—a prognosis that is worse than most cancers [6]. The morbidity associated with heart failure costs the UK National Health Service (NHS) around 2% of its annual budget, which is primarily through costs associated with hospitalisation [7]. Two percent of all hospitalised bed-days and 5% of all emergency hospital admissions are a result of heart failure [4, 8].

Depression is characterised by symptoms that affect a patient’s cognitive, emotional and behavioural processes [9]. The association between depression and heart failure has been demonstrated in numerous studies; however, the specific rate of prevalence varies, ranging from 9% [10] to as much as 60% [11], with one meta-analysis reporting a pooled estimate of 22% [12]. The existence of depression has negative implications for heart failure patients, particularly through reduced survival and an increased risk of secondary events [12–15]. The precise mechanism by which depression causes poorer outcomes in heart failure patients is unclear, but is thought to be a combination of behavioural influences and their interaction with physiological responses [16–18]. In addition, the behavioural influences of depression can reduce the likelihood of both treatment adherence [19] and modifying lifestyle behaviours [16], which may further contribute to adverse outcomes.

Cognitive behavioural therapy (CBT) refers to a group of psychological interventions that aim to understand a patient’s normal cognitive and behavioural processes, and modify these to eliminate negative cognitions and behaviours [20, 21]. CBT is a well-established intervention in depression, and is currently recommended in guidelines [22], but its effectiveness for depression in heart failure patients remains unclear [23]. Two previous systematic reviews only identified one study that had examined psychological interventions for depression in heart failure patients [3, 24]. For a condition with an already poor prognosis and reduced quality of life, the potential for further deterioration with concomitant depression needs to be addressed. This systematic review evaluated the effectiveness of CBT for heart failure patients with depression by assessing changes in depression scores, impact on quality of life (QoL) and rates of hospitalisation and mortality.

## Methods

This systematic review and meta-analysis was prospectively registered with the PROSPERO database of systematic reviews (CRD42016036146) [25] and reported in accordance with the Preferred Reporting Items for Systematic reviews and Meta-Analyses (PRISMA) guidelines [26].

### Study selection

The search strategy was developed by the research team and checked by an information specialist prior to implementation (see Supplementary material 1). The strategy included specific terms relevant to the study objectives: CBT, heart failure and depression. It was primarily developed for Ovid MEDLINE, before adaptation for use in other bibliographic databases. The following bibliographic databases were searched from inception to 6 March 2016: PubMed/MEDLINE, EMBASE, PsycINFO, Cochrane Central Register of Controlled Trials (CENTRAL) and CINAHL. Grey literature was found through contacting lead authors on heart failure for unpublished studies and through manual searches of reference lists of included papers. Two reviewers independently screened the titles, abstracts and full texts of studies, as appropriate, for potentially relevant studies. Disagreements were resolved through discussion or adjudication by a third person.

### Inclusion and exclusion criteria

Both randomised controlled trials (RCTs) and observational studies were eligible for inclusion. Studies were only included if they met the following criteria: participants were ≥18 years, with a clinical diagnosis of heart failure as defined by each study (usually relating to a combination of clinical symptoms and identification of either systolic or diastolic dysfunction) and with depression or depressive symptoms (above or equal to a predefined cut-off on validated depression questionnaires). The intervention was CBT, as described by authors, entailing both cognitive and behavioural components; studies comprising solely of cognitive or behavioural therapies were excluded, as were CBT interventions as part of a more comprehensive package (e.g. with exercise). Comparators included usual care, exercise, medication or no treatment. There were no language restrictions imposed, but accessibility of full-text publication was a requirement. Two reviewers independently determined eligibility for inclusion/exclusion, with disagreements discussed and referred to a third reviewer if required.

### Outcomes

The following outcomes were of interest: change in depression (assessed through changes to depression scores on validated questionnaires), quality of life (assessed by a validated quality of life questionnaire) and clinical outcomes of hospitalisation and mortality (both all-cause mortality and cardiovascular mortality). However, there was insufficient information on cause of death to examine the impact of CBT on cardiovascular mortality.

### Data extraction and risk of bias assessment

Two reviewers used a standardised data extraction form to independently extract the following information: study population (baseline characteristics, sample size, definition of heart failure and assessment of depression/depressive symptoms), CBT intervention (description of CBT, duration and frequency), comparator (type of comparator, duration and frequency) and the outcomes (method of assessment, depression scores, quality of life scores, number of hospitalisations and deaths). Included studies were assessed for methodological quality using risk of bias assessment tools. For RCTs, the Cochrane Risk of Bias tool [27] was used, which considers selection bias (randomisation and allocation concealment), performance bias (blinding of participants and comparators), detection bias (blinding of assessed outcomes), attrition bias (incomplete outcome data) and reporting bias. For the assessment of observational studies, the Risk of Bias Assessment tool for Non-randomised Studies (RoBANS) [28] was employed.

### Statistical analysis

The outcome data from included studies were reported as either dichotomous or continuous variables. For dichotomous data, the risk ratio and 95% confidence intervals were reported. For continuous variables, the mean differences (MD) between CBT and comparator groups were reported, with standardised mean differences (SMD) and 95% confidence intervals calculated. In studies where the mean difference was not reported, it was calculated for relevant time-points by using individual patient data obtained from the authors of individual studies. A meta-analysis of quantitative data was performed using fixed-effects modelling, under the assumption that there would be a similar effect from trials with similar patient populations and outcomes [29]. As a sensitivity analysis, we performed random effects modelling using the method of DerSimonian and Laird [30]. For outcomes measured by different questionnaires, subgroup analyses were performed separately for all questionnaires and for questionnaires which were common across included studies. All effect estimates were accompanied by 95% confidence intervals and assessed for heterogeneity (using the *I* ^2^ statistic). All statistical tests were two-tailed, and *p* values of <0.05 were considered statistically significant. Meta-analysis was performed using Review Manager (RevMan) version 5.3 (The Nordic Cochrane Centre, Copenhagen, 2014).

## Results

### Identification and selection of studies

**Fig. 1:**
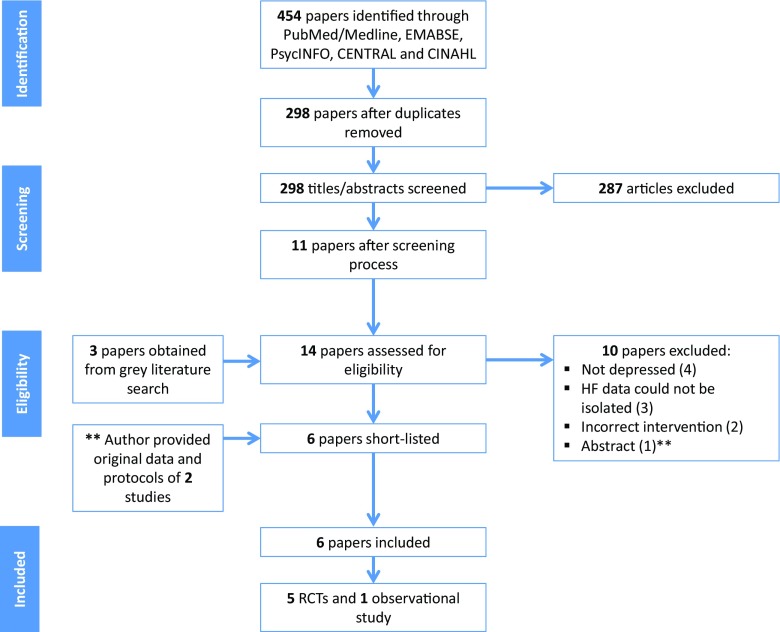
Search results and study selection process. *HF* heart failure, *RCT* randomised controlled trial

The searches revealed a total of 454 papers, reduced to 298 papers after removing duplicates (Fig. 1). After independent screening of titles and abstracts, 287 papers were determined ineligible, with 11 papers retrieved for further evaluation. An additional three papers were obtained through contacting lead authors and grey literature search, resulting in 14 papers for further evaluation. Of these, four papers were excluded due to inappropriate populations [31–34]; three papers were excluded as information on heart failure patients could not be isolated from the study population, despite contacting authors [35–37]; two papers were excluded due to inappropriate interventions [38, 39] (see Supplementary material 2). Of the remaining five papers [40–44], one was an abstract that combined data for two separate RCTs [44]; therefore, the lead author was contacted, and provided the individual patient data and corresponding protocols for the two studies (referred to as Dekker, 2010 *Personal Communication* and Dekker, 2011 *Personal Communication* from here on). Therefore, six studies were included in this systematic review.

### Description of included studies

The six studies comprised 320 participants with predominantly New York Heart Association (NYHA) classes II-III, who were mostly male (ranging from 43 to 70% across studies), with mean ages ranging from 55 to 66 years (Table 1). Five were RCTs [40–42 Dekker 2010, Dekker 2011], and one study was observational [43]. Four of the RCTs had one intervention group whilst one [42] had three intervention groups (one was a combination of CBT with exercise and was excluded from these analyses). In all the RCTs, the CBT intervention consisted of individual, face-to-face sessions that were delivered by an experienced nurse or therapist. These sessions varied in frequency and duration: three studies had single sessions each lasting 30 min [40, Dekker 2010, Dekker 2011]; one study had weekly sessions lasting 60 min for 12 weeks [42]; and one study initially had 60-min weekly sessions, before tapering to bi-weekly and then bi-monthly sessions, for a total of 6 months [41]. The CBT sessions generally involved building rapport with patients, understanding patient thoughts and behaviours about heart failure, educating patients about depression and setting and reviewing assignments. For all RCTs, the face-to-face CBT sessions were followed up by ‘booster’ CBT telephone sessions. The comparator in the RCTs was usual care only, with one study having a second, exercise only comparator group. The observational study was a proof-of-concept study, which consisted of an Internet-based programme that delivered CBT modules over a 9-week period, with contact with healthcare professionals only through written feedback on assignments. Attrition rates were low across studies, except for two studies [Dekker 2010; Dekker 2011], where there were significant losses to follow-up beyond the 1 week time-point (Table 1).

**Table 1:** Characteristics of the included studies comparing the effect of CBT versus usual care or exercise on depression in heart failure patients

Study author; year; country; type	Sample size (*n*); mean (SD) age; % female	% NYHA classes III-IV; mean (SD) LVEF	Setting	Depression cut-off for inclusion	CBT intervention (main phase and additional phase)	Comparator(s)	Outcomes (modality used)^b^	Follow-up^c^ (participants after attrition, *n*)
Gary 2010 USA RCT	*n* = 56^a^ 65.8 years (13.5) 57.1	56.7 ^d^	Outpatient	HAM-D score > 11	Weekly, 60 min, face-to-face CBT sessions delivered by nurse (for 3 months); weekly, then bi-monthly telephone sessions (for 3 months)	Usual care, exercise, CBT and exercise	Depression (BDI-II, HAM-D) QoL (MLHFQ) Mortality	1 month (^d^) 2 months (^d^) *3 months* (*n* = 52) 6 months (*n* = 47)
Dekker 2010 USA RCT	*n* = 28 56.1 years (11.1) 50	57.1 37.9 (16.6)	Hospital	PHQ-9 score > 10	One, 30 min, face-to-face CBT session delivered by nurse; 1× additional telephone session	Usual care	Depression (BDI-II, PHQ-9) QoL (MLHFQ) Hospitalisations Mortality	*1 week* (*n* = 15) 3 months (*n* = 10) 6 months (*n* = 6)
Dekker 2011 USA RCT	*n* = 30 55.6 years (9.8) 30	60 30.6 (13.1)	Hospital	PHQ-9 score > 5	One, 30 min, face-to-face CBT session delivered by nurse; 4× additional telephone sessions	Usual care	Depression (BDI-II, PHQ-9) QoL (MLHFQ) Hospitalisations Mortality	*1 week* (*n* = 18) 3 months (*n* = 11)
Dekker 2012 USA RCT	*n* = 41 66 years (11.0) 45	81 39.5 (16.4)	Hospital	BDI-II score 10–28	One, 30 min, face-to-face CBT session delivered by nurse; 1× additional telephone session	Usual care	Depression (BDI-II) QoL (MLHFQ) Hospitalisations Mortality	*1 week* (*n* = 38) 3 months (*n* = 34)
Freedland 2015 USA RCT	*n* = 158 55.8 years (11.2) 46.2	42.4 38.9 (15.5)	Outpatient	BDI-II score ≥ 14	Weekly or bi-weekly, 60 min, face-to-face CBT session delivered by therapist (for 6 months); 4× additional telephone sessions (for 6 months)	Usual care	Depression (BDI-II, HAM-D) QoL (KCCQ, SF-12) Hospitalisations	3 months (*n* = 138) *6 months* (*n* = 132) 9 months (*n* = 119) 12 months (*n* = 119)
Lundgren 2015 Sweden Observational	*n* = 7 62 years (10.0) 57.1	^d^ ^d^	Outpatient	PHQ-9 score > 5	Internet-based CBT sessions delivered by a programme over 9 weeks	^d^	Depression (PHQ-9, MADRS)	9 weeks (*n* = 6)

*BDI-II* Beck’s Depression Inventory-revised, *CBT* cognitive behavioural therapy, *HAM-D* Hamilton Rating Scale for Depression, *KCCQ* Kansas City Cardiomyopathy Questionnaire, *LVEF* left ventricular ejection fraction, *MADRS* Montgomery-Åsberg Depression Rating Scale, *MLHFQ* Minnesota Living with Heart Failure Questionnaire, *n* number, *NYHA* New York Heart Association, *PHQ-9* Patient Health Questionnaire, *QoL* quality of life, *RCT* randomised controlled trial, *SD* standard deviation, *SF-12* Short Form-12

^a^Number of participants excluding CBT and exercise group

^b^Outcomes only listed if appropriate for this review

^c^Follow-up points in italics are the first time-points after the face-to-face CBT sessions

^d^Not reported

### Risk of bias

**Fig. 2:**
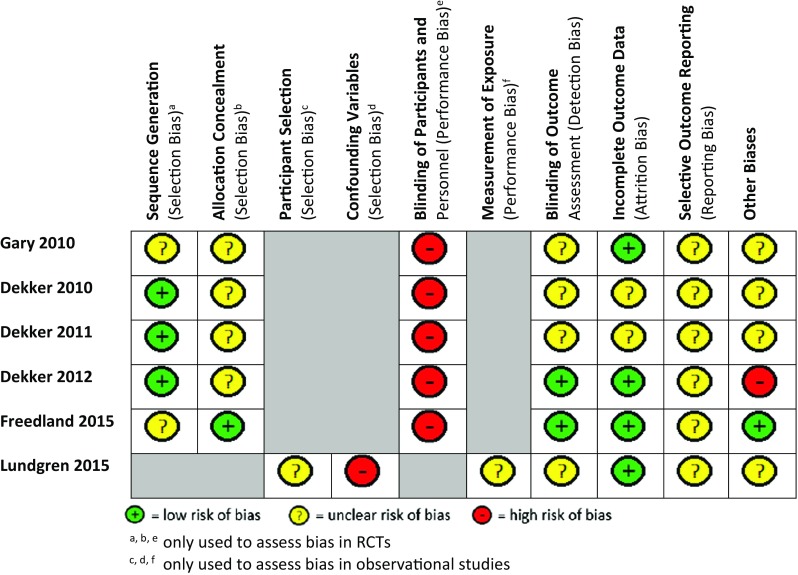
Risk of bias assessment. Risk of bias was performed using the Cochrane Risk of Bias tool for RCTs and the Risk of Bias Assessment tool for Non-randomised Studies (RoBANS) for observational studies

For the five RCTs, the process of randomisation was described in adequate detail for three studies [40 Dekker 2010, Dekker 2011], but for two studies, the precise method of random sequence generation was not clear [41, 42]. Allocation concealment was only explained thoroughly in Freedland et al. [41]. Given the nature of the CBT intervention, blinding of participants was not possible; only two studies sufficiently explained the blinding of outcome assessors [40, 41] (Fig. 2). Incomplete outcome data was addressed in three RCTs with appropriate reasons provided for attrition and similar rates between groups [40–42]. Selective reporting was difficult to ascertain due to a lack of published protocols. The RCTs were free from other sources of bias, except for one study [40] with potential social desirability bias due to the follow-up questionnaires being administered by the same nurse that delivered the intervention. For the observational study, the nature of the CBT intervention could have resulted in sampling bias, as patients required access to the Internet and basic computer skills. Further, the level of exposure participants had to the intervention is unclear, as no healthcare professionals were present during the intervention itself. Some potential confounding variables were not included (i.e. severity of heart failure), but there were no signs of attrition bias or selective outcome reporting. Small study bias could not be assessed by testing for funnel plot asymmetry as there were <10 studies [29] in this meta-analysis.

### Outcomes

**Fig. 3:**
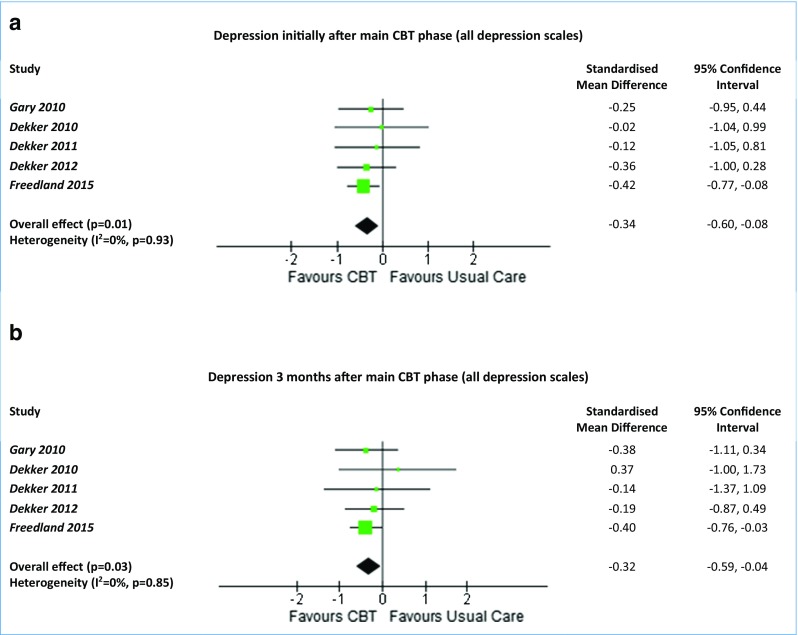
Forest plots summarising the effectiveness of CBT versus usual care on depression. Meta-analysis of all depression scales (BDI-II and HAM-D) at the first time-point initially after the main CBT phase (**a**) and at 3 months (**b**). *CBT* cognitive behavioural therapy

For depression and quality of life, the effects of CBT were evaluated at two time-points: the first time-point was after the main CBT phase and again 3 months later, due to the differing lengths of CBT and follow-up points in each trial (Table 1; Fig. 3).

#### Depression

The change in depressive symptoms was assessed by depression scores on validated questionnaires. All the RCTs used mean Beck’s Depression Inventory (BDI-II) scores, except one [41], which used the Hamilton Rating Scale for Depression (HAM-D); some also used an additional depression questionnaire (HAM-D or Patient Health Questionnaire (PHQ-9) [41, 42 Dekker 2010, Dekker 2011]. The observational study [43] used the PHQ-9 and Montgomery-Åsberg Depression Rating Scale (MADRS) and was not included in the meta-analysis. This study [43] demonstrated a decrease in median depression scores from baseline (PHQ-9 11, MADRS 25.5) to 9 weeks (PHQ-9 8.5, MADRS 16.5). Similarly, all RCTs demonstrated improvement in the mean depression scores in both intervention and comparator groups when compared to baseline depression scores at any of the follow-up points [40–42, Dekker 2010, Dekker 2011] (Table 2).

**Table 2:** Meta-analysis results for depression, quality of life, mortality and hospitalisations

Outcome	Number of studies	Number of participants	Statistical method used	Effect estimate, mean, risk ratio (95% CI)	*p* value	Heterogeneity, *I* ^2^ (%)
Depression (initially after CBT)						
BDI-II	4	203	Standardised mean difference (fixed effects)	−0.35 (−0.63 to −0.07)	0.01	0
All scales	5	235	Standardised mean difference (fixed effects)	−0.34 (−0.60 to −0.08)	0.01	0
Depression (3 months after CBT)						
BDI-II	4	174	Standardised mean difference (fixed effects)	−0.30 (−0.61 to −0.00)	0.05	0
All scales	5	204	Standardised mean difference (fixed effects)	−0.32 (−0.59 to −0.04)	0.03	0
QoL (initially after CBT)						
MLHFQ	4	88	Standardised mean difference (fixed effects)	−0.25 (−0.68 to 0.18)	0.26	52
All scales	5	220	Standardised mean difference (fixed effects)	−0.31 (−0.58 to −0.05)	0.02	38
QoL (3 months after CBT)						
MLHFQ	4	78	Standardised mean difference (fixed effects)	−0.13 (−0.58 to 0.33)	0.58	0
All scales	5	197	Standardised mean difference (fixed effects)	−0.22 (0.51 to 0.06)	0.12	0
Mortality						
All-cause mortality	4	135	Risk ratio (fixed effects)	1.05 (0.44 to 2.52)	0.92	0
Hospitalisations						
All-cause hospitalisations	4	257	Risk ratio (fixed effects)	0.99 (0.75 to 1.32)	0.96	0

*BDI-II* Beck Depression Inventory-revised, *CBT* cognitive behavioural therapy *CI* confidence intervals, *MLHFQ* Minnesota Living with Heart Failure Questionnaire, *QoL* quality of life

Immediately after completion of the main CBT phase, there was a greater improvement in depression for the CBT group compared to the usual care group for BDI-II scores (SMD −0.35, 95% CI −0.63 to −0.07, *p* = 0.01, *I* ^2^ = 0%) and across all depression scales (SMD −0.34, 95% CI −0.60 to −0.08, *p* = 0.01, *I* ^2^ = 0%) (Fig. 3a). Three months after the main CBT phase, there was still a greater improvement in the CBT group than in the usual care group for BDI-II scores (SMD −0.30, 95% CI −0.61 to −0.00, *p* = 0.05, *I* ^2^ = 0%) and across all depression scales (SMD −0.32, 95% CI −0.59 to −0.04, *p* = 0.03, *I* ^2^ = 0%) (Fig. 3b). This demonstrates a moderate size effect initially after the main CBT phase which was maintained 3 months later, and there was no evidence of heterogeneity (*I* ^2^ = 0%). For the one study [42] that compared CBT to exercise, there was no difference in depression at 3 months for HAM-D scores (MD −0.34, 95% CI −4.93 to 4.24).

#### Quality of life

**Fig. 4:**
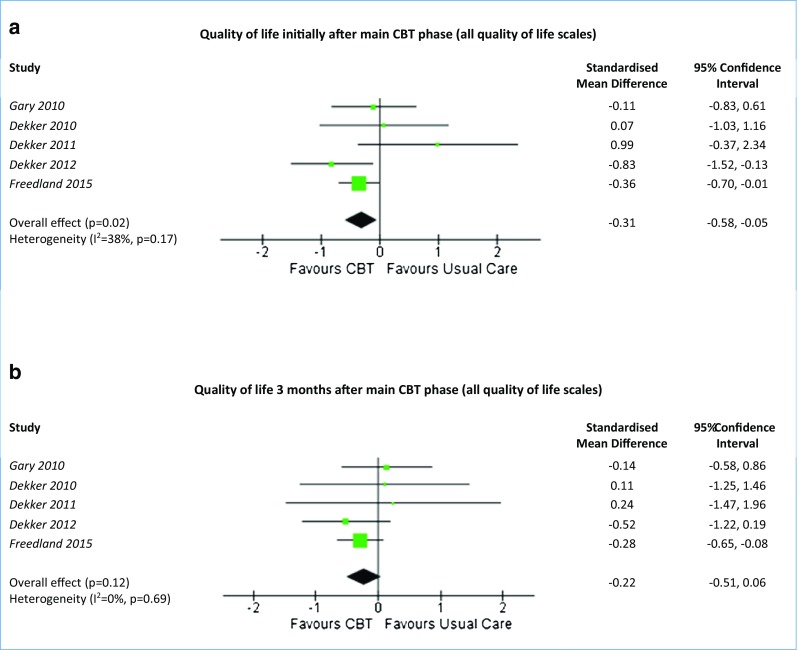
Forest plots summarising the effectiveness of CBT versus usual care on quality of life. Meta-analysis of all quality of life scales (MLHFQ and KCCQ) at the first time-point initially after the main CBT phase (**a**) and at 3 months (**b**). *CBT* cognitive behavioural therapy

Quality of life was only assessed in the RCTs. Four studies used the Minnesota Living with Heart Failure Questionnaire (MLHFQ) [40, 42, Dekker 2010, Dekker 2011], whilst Freedland et al. [41] used both the Kansas City Cardiomyopathy Questionnaire (KCCQ) and the Short Form 12-item (SF-12). There were improvements in quality of life scores for the CBT intervention groups across all five RCTs, when comparing baseline scores to any of the follow-up points. Immediately after the main CBT phase, there was no difference between the CBT group and usual care group for MLHFQ scores (SMD −0.25, 95% CI −0.68 to 0.18, *p* = 0.26, *I* ^2^ = 52%) (data not shown). However, when all QoL data were combined, there was a greater improvement in the CBT group than the usual care group (SMD −0.31, 95% CI −0.58 to −0.05, *p* = 0.02, *I* ^2^ = 38%) (Fig. 4a), although there was moderate heterogeneity (*I* ^2^ values of 38 and 52%, respectively). At 3 months, there were no differences in QoL between the CBT and the usual care groups for either MLHFQ scores (SMD −0.13, 95% CI −0.58 to 0.33, *p* = 0.58, *I* ^2^ = 0%) or across all QoL scales (SMD −0.22, 95% CI −0.51 to 0.06, *p* = 0.12, *I* ^2^ = 0%) (Fig. 4b). One study that compared CBT to exercise demonstrated no difference in QoL between the groups at 3 months (MD −4.23, 95% CI −22.24 to 13.78).

#### Clinical outcomes

There were limited data on clinical outcomes, and hence, we have limited power for comparison. Four studies provided data on all-cause mortality [40, 42, Dekker 2010, Dekker 2011], with 8/69 (11.6%) deaths in CBT and 7/66 (10.6%) deaths in usual care. There was no difference in all-cause mortality and no evidence of heterogeneity (risk ratio 1.05, 95% CI 0.44 to 2.52, *p* = 0.63, *I* ^2^ = 0%).

Hospitalisations were reported in four studies [40, 41, Dekker 2010, Dekker 2011]. Overall, 55/129 (42.6%) and 55/128 (43.0%) patients in the CBT and usual care groups were hospitalised, respectively, with no difference between the two groups and no evidence of heterogeneity (risk ratio 0.99, 95% CI 0.75 to 1.32, *p* = 0.96, *I* ^2^ = 0%).

## Discussion

This systematic review and meta-analysis suggests that CBT may be more effective than usual care at improving depression in heart failure patients initially after the CBT sessions. This difference was sustained 3 months after completion of the CBT sessions; however, these were highly selected patients in selected centres with varied comparators and subjective outcome measures (depression and QoL). The improvement in depression scores evident at 3 months was greater in two RCTs [41, 42]. This may be due to the frequency and duration of the CBT, which were weekly and over a period of time [41, 42], as opposed to a single CBT session [40, Dekker 2010, Dekker 2011]. For quality of life, CBT showed a greater improvement when compared to usual care initially after the main CBT phase; however, there was no evidence of a difference in QoL between the two groups at subsequent time-points. There was no evidence of CBT having an effect on either hospitalisation or mortality.

This systematic review was conducted to evaluate the effects of CBT on depression, quality of life, hospitalisation and mortality in heart failure patients. Previously, there have been two systematic reviews [3, 24] that evaluated the effects of psychological interventions on depression in heart failure patients. However, the first, a 2005 Cochrane review on psychological interventions for depression in heart failure [3], found no relevant RCTs, highlighting the need for RCTs on psychological interventions to be conducted. The second, a 2012 systematic review on the effects of interventions on depression in heart failure, only identified one RCT with a CBT intervention, concluding that there was insufficient evidence on the effects of CBT [24]. Therefore, the emergence of new RCTs, and the lack of conclusive evidence from previous systematic reviews on this topic, justifies the need for the current systematic review to evaluate the effects of CBT in heart failure patients.

Despite the findings of this systematic review, there are several limitations that need to be acknowledged. The searches identified only five RCTs, which demonstrate the lack of experimental studies assessing the effects of CBT in heart failure patients. Two of the RCTs had small sample sizes (≤30), which were particularly problematic with longer follow-up due to attrition. There was also a lack of studies that evaluated the effects of CBT with follow-up ≥6 months, which would have been useful in assessing the long-term sustainability of the effects of CBT. Overall, the methodological quality of studies was unclear due to insufficient information provided. For two studies, only the original data and study protocols could be accessed [Dekker 2010; Dekker 2011], which led to a lack of clarity over certain risks of biases. There was performance bias in the RCTs due to a lack of blinding of participants, but this was unavoidable due to the nature of CBT interventions. Two outcome measures (depression and QoL) were subjective and assessed by self-reported questionnaires, which may have introduced social desirability bias. Data on hospitalisations and deaths were limited and were not reported in all studies. There was also insufficient data to determine the relative effects of CBT in comparison to exercise for depression in heart failure patients, as there was only one RCT that utilised exercise as a comparator. One advantage of this review, however, was that additional data was obtained by contacting lead authors to better inform the systematic review and enable the meta-analysis. Future RCTs on the effects of CBT for heart failure patients would benefit from recruiting larger numbers of participants, delivering weekly CBT sessions over a longer period of time, with long-term follow-up (≥6 months), and reporting on outcomes such as hospitalisations or mortality.

Current NICE guidelines on chronic heart failure [45] state that depression should be treated in accordance with the guidelines for adults with depression [22] and those for adults with a chronic physical health problem [46]; however, the evidence used to evaluate the effects of CBT on depression are not specific to heart failure patients. This may be due to the lack of randomised controlled trials on CBT for depression in heart failure prior to the publication of these guidelines. The 2016 Scottish Intercollegiate Guidelines Network (SIGN) guidelines on the management of chronic heart failure offer a conditional recommendation that CBT should be considered in heart failure patients with depression [47]. The evidence for this recommendation is based on a single RCT on CBT and a systematic review on interventions [24] that included only a single CBT intervention study. Therefore, the current systematic review and meta-analysis extends previous work and provides a comprehensive review of available evidence for the effects of CBT for depression in heart failure patients. The findings from this research have identified an area of further study in heart failure and demonstrated the potential of CBT in selected research centres.

## Conclusion

CBT may be more effective than usual care at improving depression in heart failure patients initially after the main CBT phase and 3 months afterwards. CBT also appears to be more effective than usual care at improving quality of life in heart failure patients initially after the main CBT phase, but this effect was not sustained 3 months later. There were no observable differences between CBT and usual care for hospitalisations or mortality. However, these findings were limited by the small sample sizes in some studies, lack of long-term follow-up, use of subjective outcome measures and insufficient information to assess methodological quality. Larger and more robust RCTs are needed to ascertain the long-term benefits and cost-effectiveness of a CBT intervention for depression in heart failure patients.

## Electronic supplementary material

Supplementary Table 1 (DOCX 24 kb).

Supplementary Table 2 (DOCX 23 kb).

Supplementary Table 3 (DOCX 26 kb).
