# Supplementary material for: Effects of cognitive behavioural therapy for depression in heart failure patients: a systematic review and meta-analysis
Source: Heart Fail Rev. 2017 Jul 22;22(6):731–41. doi: 10.1007/s10741-017-9640-5 (PMC5635071; doi:10.1007/s10741-017-9640-5)
Supplement: Supplementary file 1 — (DOCX 24 kb). [file 10741_2017_9640_MOESM1_ESM.docx]

**Supplementary Materials 1: Search Strategy**

| *Ovid MEDLINE*   1. exp HEART FAILURE 2. (heart failure ab/kf/ot/ti) 3. (cardiac failure ab/kf/ot/ti) 4. exp VENTRICULAR DYSFUNCTION 5. (ventricular dysfunction ab/kf/ot/ti) 6. #1 or 2# or 3# or 4# or 5# 7. exp COGNITIVE THERAPY/ or exp BEHAVIOR THERAPY/ 8. (cognitive adj6 therapy ab/kf/ot/ti) 9. (cognitive adj6 therapist ab/kf/ot/ti) 10. (cognitive adj6 therapies ab/kf/ot/ti) 11. (cognitive adj6 therapists ab/kf/ot/ti) 12. (behavioural adj6 therapy ab/kf/ot/ti) 13. (behavioural adj6 therapies ab/kf/ot/ti) 14. (behavioural adj6 therapist ab/kf/ot/ti) 15. (behavioural adj6 therapists ab/kf/ot/ti) 16. (behavioral adj6 therapy ab/kf/ot/ti) 17. (behavioral adj6 therapies ab/kf/ot/ti) 18. (behavioral adj6 therapist ab/kf/ot/ti) 19. (behavioral adj6 therapists ab/kf/ot/ti) 20. (behaviour adj6 therapy ab/kf/ot/ti) 21. (behaviour adj6 therapies ab/kf/ot/ti) 22. (behaviour adj6 therapist ab/kf/ot/ti) 23. (behaviour adj6 therapists ab/kf/ot/ti) 24. (behavior adj6 therapy ab/kf/ot/ti) 25. (behavior adj6 therapies ab/kf/ot/ti) 26. (behavior adj6 therapist ab/kf/ot/ti) 27. (behavior adj6 therapists ab/kf/ot/ti) 28. #7 or #8 or #9 or #10 or #11 or #12 or #13 or #14 or #15 or #16 or #17 or #18 or #19 or #20 or #21 or #22 or #23 or #24 or #25 or #26 or #27 29. exp DEPRESSION 30. exp DEPRESSIVE DISORDER 31. (depress* ab/kf/ot/ti) 32. #29 or #30 or #31 33. #6 AND #28 AND #32   MeSH terms are in capitals and textwords are in lower case | *PsycINFO*   1. HEART FAILURE 2. (heart failure ab/hw/id/ot/ti) 3. (cardiac failure ab/hw/id/ot/ti) 4. VENTRICULAR DYSFUNCTION 5. (ventricular dysfunction ab/hw/id/ot/ti) 6. #1 or 2# or 3# or 4# or 5# 7. exp COGNITIVE THERAPY/ or exp COGNITIVE BEHAVIOR THERAPY/ 8. exp BEHAVIOR THERAPY/ 9. (cognitive adj6 therapy ab/hw/id/ot/ti) 10. (cognitive adj6 therapies ab/hw/id/ot/ti) 11. (cognitive adj6 therapist ab/hw/id/ot/ti) 12. (cognitive adj6 therapists ab/hw/id/ot/ti) 13. (behavioural adj6 therapy ab/hw/id/ot/ti) 14. (behavioural adj6 therapies ab/hw/id/ot/ti) 15. (behavioural adj6 therapist ab/hw/id/ot/ti) 16. (behavioural adj6 therapists ab/hw/id/ot/ti) 17. (behavioral adj6 therapy ab/hw/id/ot/ti) 18. (behavioral adj6 therapies ab/hw/id/ot/ti) 19. (behavioral adj6 therapist ab/hw/id/ot/ti) 20. (behavioral adj6 therapists ab/hw/id/ot/ti) 21. (behaviour adj6 therapy ab/hw/id/ot/ti) 22. (behaviour adj6 therapies ab/hw/id/ot/ti) 23. (behaviour adj6 therapist ab/hw/id/ot/ti) 24. (behaviour adj6 therapists ab/hw/id/ot/ti) 25. (behavior adj6 therapy ab/hw/id/ot/ti) 26. (behavior adj6 therapies ab/hw/id/ot/ti) 27. (behavior adj6 therapist ab/hw/id/ot/ti) 28. (behavior adj6 therapists ab/hw/id/ot/ti) 29. #7 or #8 or #9 or #10 or #11 or #12 or #13 or #14 or #15 or #16 or #17 or #18 or #19 or #20 or #21 or #22 or #23 or #24 or #25 or #26 or #27 or #28 30. exp DEPRESSION (emotion)/ or exp Major Depression 31. (depress* ab/hw/id/ot/ti) 32. #30 or #31 33. #6 AND #29 AND #32 |
| --- | --- |

| *PubMed*   1. HEART FAILURE 2. (heart failure ab/ti) 3. (cardiac failure ab/ti) 4. VENTRICULAR DYSFUNCTION 5. (ventricular dysfunction ab/ti) 6. #1 OR #2 OR #3 OR #4 OR #5 7. COGNITIVE THERAPY 8. BEHAVIOR THERAPY 9. (cognit* therap* ab/ti) 10. (behavio* therap* ab/ti) 11. (cognit* adj6 therap* ab/ti) 12. (behavio* adj6 therap* ab/ti) 13. #7 OR #8 OR #9 OR #10 OR #11 OR #12 14. DEPRESSION 15. DEPRESSIVE DISORDER 16. (depress* ab/ti) 17. #14 OR #15 OR #16 18. #6 AND #13 AND #17   *CINAHL*   1. (heart failure ti/ab) 2. (cardiac failure ti/ab) 3. (ventricular dysfunction ti/ab) 4. #1 or 2# or 3# 5. COGNTIIVE THERAPY/ or BEHAVIOR THERAPY/ or COGNITIVE THERAPY (lowa NIC)/ or BEHAVIOR THERAPY (lowa NIC) 6. (cognitive therap* ti/ab) 7. (behavi therap* ti/ab) 8. #5 or #6 or #7 9. DEPRESSION 10. (depress* ti/ab) 11. #9 or #10 12. #4 AND #8 AND #11   *CENTRAL*   1. exp HEART FAILURE 2. (heart next failure ti/ab/kw) 3. (cardiac next failure ti/ab/kw) 4. exp VENTRICULAR DYSFUNCTION 5. (ventricular next dysfunction ti/ab/kw) 6. #1 or #2 or #3 or #4 or #5 7. exp COGNITIVE THERAPY 8. exp BEHAVIOR THERAPY 9. (cognitive near therap* ti/ab/kw) 10. (behavio* near therap* ti/ab/kw) 11. (CBT ti/ab/kw) 12. #7 or #8 or #9 or #10 or #11 13. exp DEPRESSION 14. exp DEPRESSIVE DISORDER 15. (depress* ti/ab/kw) 16. #13 or #14 or #15   #6 AND #12 AND #16 | *EMBASE*   1. exp HEART FAILURE 2. (heart failure ti/ot/hw/ab/kw) 3. (cardiac failure ti/ot/hw/ab/kw) 4. (ventricular dysfunction ti/ot/hw/ab/kw) 5. #1 or 2# or 3# or 4# 6. COGNTIIVE BEHAVIOURAL THERAPY mp/ or exp COGNITIVE THERAPY 7. exp BEHAVIOR THERAPY/ 8. (cognitive adj6 therapy ti/ot/hw/ab/kw) 9. (cognitive adj6 therapies ti/ot/hw/ab/kw) 10. (cognitive adj6 therapist ti/ot/hw/ab/kw) 11. (cognitive adj6 therapists ti/ot/hw/ab/kw) 12. (behavioural adj6 therapy ti/ot/hw/ab/kw) 13. (behavioural adj6 therapies ti/ot/hw/ab/kw) 14. (behavioural adj6 therapist ti/ot/hw/ab/kw) 15. (behavioural adj6 therapists ti/ot/hw/ab/kw) 16. (behavioral adj6 therapy ti/ot/hw/ab/kw) 17. (behavioral adj6 therapies ti/ot/hw/ab/kw) 18. (behavioral adj6 therapist ti/ot/hw/ab/kw) 19. (behavioral adj6 therapists ti/ot/hw/ab/kw) 20. (behaviour adj6 therapy ti/ot/hw/ab/kw) 21. (behaviour adj6 therapies ti/ot/hw/ab/kw) 22. (behaviour adj6 therapist ti/ot/hw/ab/kw) 23. (behaviour adj6 therapists ti/ot/hw/ab/kw) 24. (behavior adj6 therapy ti/ot/hw/ab/kw) 25. (behavior adj6 therapies ti/ot/hw/ab/kw) 26. (behavior adj6 therapist ti/ot/hw/ab/kw) 27. (behavior adj6 therapists ti/ot/hw/ab/kw) 28. #6 or #7 or #8 or #9 or #10 or #11 or #12 or #13 or #14 or #15 or #16 or #17 or #18 or #19 or #20 or #21 or #22 or #23 or #24 or #25 or #26 or #27 29. exp DEPRESSION 30. (depress* ti/ot/hw/ab/kw) |
| --- | --- |
