# Supplementary material for: Effects of cognitive behavioural therapy for depression in heart failure patients: a systematic review and meta-analysis
Source: Heart Fail Rev. 2017 Jul 22;22(6):731–41. doi: 10.1007/s10741-017-9640-5 (PMC5635071; doi:10.1007/s10741-017-9640-5)
Supplement: Supplementary file 2 — (DOCX 23 kb). [file 10741_2017_9640_MOESM2_ESM.docx]

**Supplementary Materials 2: Table of Excluded Studies**

| Study ID; Type | Sample size | Participants | Intervention | Outcomes |
| --- | --- | --- | --- | --- |
| Berkman 2003 RCT | 2481 | Not exclusively HF patients (334 with HF), unable to isolate HF patients | CBT plus selective serotonin reuptake inhibitor when indicated (HAM-D > 24) | Composite primary endpoint of death or recurrent myocardial infarction |
| Carney 2016  Observational | 157 | Not exclusively HF patients (35 with HF), unable to isolate HF patients | Participants were given CBT, and if not meeting targets were also started on antidepressants | Cardiac risk markers, depression, physical activity |
| Chung 2014  RCT (abstract) | 24 dyads | Patients with HF, along with their caregivers (recruited in dyads) | Combination of CBT and educational self-care | Perceived control, depression, quality of life |
| Cockayne 2014 RCT | 260 | Participants did not have to be depressed for inclusion | Individual CBT sessions with nurse | All-cause hospitalisations, quality of life, depression |
| Cully 2010  Observational | 23 | Participants had either anxiety or depression, with HF or COPD (unable to isolate HF and depressed) | Individual CBT sessions with psychologist | Depression, anxiety, COPD disease-specific outcomes, quality of life |
| Moser 2012  RCT (abstract) | 280 | Participants with HF, but with or without depression | CBT and relaxation therapy by nurse practitioner | Depression, quality of life |
| Redeker 2013  RCT (abstract) | 46 | Patients with HF and insomnia (did not have to be depressed for inclusion) | CBT targeting insomnia | Insomnia, fatigue, sleepiness, depression, anxiety |
| Redeker 2015  RCT | 52 | Patients with HF and insomnia (did not have to be depressed for inclusion) | CBT targeting insomnia | Feasibility of CBT for insomnia, daytime symptoms, fatigue, depression |
| Smeulders 2010  RCT | 317 | Patients with HF but depression/depressive symptoms not an inclusion criteria | CBT session delivered by cardiac nurse | Self-efficacy, perceived control, self-care behaviour, quality of life, depression |

CBT (Cognitive Behavioural Therapy); HAM-D (Hamilton Rating Scale for Depression); HF (Heart Failure); RCT (Randomised Controlled Trial)
